# Infection and quality of life after local or full scalp shaving in microsurgical treatment for unruptured intracranial aneurysm sited in anterior circulation

**DOI:** 10.1186/s41016-026-00449-3

**Published:** 2026-09-16

**Authors:** Yifei Hou, Yinghe Feng, Jing Zhang, Kaige Zheng, Jinhao Zhang, Qingyuan Liu, Peng Liu, Yan Zhang, Jun Wu, Shuo Wang, Donghong Zhao, Siyuan Chen

**Affiliations:** Department of Neurosurgery, Beijing Tiantan Hospital, China National Clinical Research Center for Neurological Diseases, Capital Medical University, Beijing, China

**Keywords:** Unruptured intracranial aneurysms, Microsurgical treatment, Preoperative scalp shaving, Infection, Quality of life

## Abstract

**Background:**

Scalp shaving constitutes a crucial preoperative preparation measure for the microsurgical treatment of unruptured intracranial aneurysms (UIAs) located in the anterior circulation. This study aimed to investigate the infection and quality of life after local scalp shaving (LSS) or full scalp shaving (FSS) in microsurgery for UIAs in the anterior circulation.

**Methods:**

This study included patients receiving microsurgical treatment for UIAs sited in the anterior circulation from a national, multicenter, prospective cohort study, conducted from December 2021 to December 2024. Propensity score matching was performed to form a nested case–control cohort regarding age, sex, diabetes mellitus, aneurysm site, and size, and surgical methods with a ratio of patients with LSS to patients with FSS as 1:2. The primary outcome was central nervous system infection within 90 days after surgery, and the secondary outcome was the Face-Q score on the 180th day after surgery. Logistic analysis was performed to compare the incidence of outcomes between LSS and FSS groups.

**Results:**

This study finally included 2157 patients receiving microsurgical treatment for UIAs in the anterior circulation, with 908 males and a median age of 59 years. 719 patients were assigned to the LSS group and 1438 to the FSS group. No significant difference was found in the 90-day central nervous system infection (25 of 719 [3.5%] *vs.* 49 of 1438 [3.4%]; odds ratio, 0.98; 95%CI, 0.60–1.60; *P* = 0.933). Patients in the LSS group had a higher 180-day Face-Q score compared with patients in the FSS group (4 *vs.* 2; ordinary odds ratio, 0.43; 95%CI, 0.40–0.47; *P* < 0.001).

**Conclusion:**

LSS is an appropriate preoperative scalp shaving protocol for patients receiving microsurgical treatment of UIAs sited in the anterior circulation, with no increasing risk of infection and improved quality of life after surgery.

**Trial registration:**

ChTUIA cohort (China Treatment Trial for Unruptured Intracranial Aneurysm, unique identifier: NCT05844163, Clinicaltrials.gov registry)

**Supplementary Information:**

The online version contains supplementary material available at https://doi.org/10.1186/s41016-026-00449-3.

## Background

Scalp shaving is an important preoperative preparation in microsurgical treatment for unruptured intracranial aneurysms (UIAs) in the anterior circulation. The elimination of hair within the surgical field has long been considered indispensable for the preservation of a sterile operative milieu, thereby contributing to the reduction of the risk of secondary wound infections [1, 2]. However, full scalp shaving (FSS) before surgery often induces psychological distress among patients, who may experience emotional resistance but feel compelled to comply [3]. This practice exacerbates preoperative anxiety and surgical fear, particularly among young patients and women [4]. Previous studies indicate that the presence or absence of hair has no significant correlation with surgical site infection rates [5–7]. An increasing number of neurosurgeons have begun to explore local scalp shaving (LSS), and an alternative approach where only minimal hair is removed along the incision line, which aims to reduce patient distress while preserving surgical sterility and outcomes.^7^ Notwithstanding these advancements, there remains a dearth of exhaustive data that directly juxtaposes LSS and FSS in terms of infection rates, surgical outcomes, and postoperative quality of life.

In this study, we conducted a nested case–control study based on UIA patients from a national, multicenter, prospective cohort study, aimed to investigate the appropriate preoperative scalp shaving protocol for patients receiving microsurgical treatment of UIAs sited in anterior circulation.

## Methods

### Study population

The ChTUIA cohort (unique identifier: NCT05844163), conducted from December 2021 to December 2024, was a completed national, multicenter, prospective cohort study designed to investigate appropriate management strategies for unruptured intracranial aneurysms (UIAs) [8]. This study complied with the STROBE guidelines and was approved by the institutional review board. Written informed consent was obtained from all participants or their guardians.

The present study was a matched comparative analysis nested within the prospective ChTUIA cohort. Patients who underwent microsurgical treatment in the ChTUIA cohort at 83 participating centers between December 2021 and December 2022 were screened. The inclusion criteria were: (1) age > 18 years; and (2) UIAs located in the anterior circulation and treated by microsurgery. The exclusion criteria were: (1) previous subarachnoid hemorrhage or intracerebral hemorrhage; (2) recurrent, fusiform, dissecting, traumatic, bacterial, or atrial myxomatous aneurysms; (3) a family history of intracranial aneurysms or polycystic kidney disease; (4) poor general condition with an expected survival of < 12 months; and (5) refusal of follow-up or inability to communicate because of severe mental illness. All patients were followed up for 180 days after surgery. After follow-up, the following patients were further excluded: (1) those with incomplete clipping who required further surgical treatment within a short period; and (2) those with postoperative cerebrovascular ischemic or bleeding events during hospitalization who required craniotomy or craniectomy.

### Propensity score matching process

To minimize the effects of baseline confounding, propensity score matching (PSM) was performed to construct the primary matched cohort for comparing infection and quality of life outcomes between the local scalp shaving (LSS) group and the full scalp shaving (FSS) group. The propensity score was estimated using a logistic regression model based on age, sex, diabetes mellitus, aneurysm site, aneurysm size, and surgical method (clipping or wrapping). Nearest-neighbor matching without replacement was performed in a fixed 1:2 ratio (LSS:FSS) with a caliper width of 0.1. Covariate balance after matching was assessed using standardized mean differences (SMDs), and an SMD < 0.1 was considered indicative of acceptable balance. After matching, a multivariable logistic regression model was used to further assess the association between scalp shaving protocol and 90-day central nervous system (CNS) infection.

### Preoperative scalp shaving

Prespecified definitions of LSS and FSS were applied across participating centers, although minor center-level variations in local implementation may have existed in routine clinical practice.

For patients in the FSS group, the entire scalp was shaved with sterile clippers to ensure comprehensive preparation of the surgical site. After shaving, the scalp was cleansed with antiseptic soap, dried with sterile towels, and disinfected with povidone-iodine solution. Any residual hair was meticulously removed to avoid contamination.

For patients in the LSS group, only a strip of hair along the incision line, extending from the natural hairline to 2 cm posterior to it, was shaved. The shaved area was similarly cleaned with antiseptic soap, dried, and disinfected with povidone-iodine solution. The remaining unshaved hair was styled and secured using surgical wax or hairnets to avoid interference with the sterile field. Before final disinfection, a sterile surgical drape was applied to cover the unshaved hair.

In general, the incision length for microsurgical treatment of UIAs (clipping or wrapping) was 5–10 cm.

### Follow-up and outcomes

The index date was defined as the date of microsurgical treatment for the target UIA, and all follow-up intervals were calculated from the date of surgery. All patients were followed up by outpatient visit at 90 ± 14 days after surgery and by telephone interview at 180 ± 14 days after surgery. At least three telephone numbers were recorded to minimize loss to follow-up. Outcomes were assessed by trained clinical research coordinators using prespecified definitions and standardized criteria. Because of the observational design, outcome assessors were not formally blinded to the scalp shaving protocol.

The primary outcome was the incidence of CNS infection within 90 days after surgery. This event was suspected when clinical symptoms such as fever, headache, altered mental status, seizures, or focal neurological deficits suggested possible infection. The diagnosis was confirmed by laboratory findings from two consecutive cerebrospinal fluid samples obtained on different days, including elevated white blood cell count, low glucose level, high protein level, or positive microbiological culture, as well as imaging evidence of abscess, empyema, or other CNS infectious processes.

The secondary outcomes included: (1) the incidence of incision infection within 90 days after surgery, defined as the presence of clinical signs such as redness, swelling, warmth, pain, or purulent discharge at the surgical site, confirmed by positive microbiological culture or the need for antibiotic treatment; (2) the Face-Q score at 180 days after surgery. The Face-Q score was designed according to a previous study and is provided in the Supplemental Material. It consisted of two negative questions and five positive questions, with a total score ranging from − 2 to 5. Higher Face-Q scores indicated greater satisfaction with facial appearance after surgery; and (3) neurological condition at 180 days after surgery, assessed using the modified Rankin scale (mRS). An mRS score of 0–1 was defined as a good outcome and 2–5 as a poor outcome.

### Clinical data collection

Baseline data were collected from electronic medical records, including age, sex, comorbidities (hypertension, dyslipidemia, diabetes mellitus, and ischemic cerebrovascular or cardiovascular disease [ICCD], including transient ischemic attack, ischemic stroke, and coronary heart disease), aneurysm site (internal carotid artery [ICA], anterior cerebral artery or anterior communicating artery [ACA/Acom], and middle cerebral artery [MCA]), aneurysm size (< 7 mm or 7–10 mm), surgery duration, intraoperative bleeding, intraoperative transfusion, surgical method (clipping or wrapping), and mRS score on admission.

Data were collected in a standardized manner across centers and were reviewed for completeness and consistency before analysis.

### Statistical analysis

No formal a priori sample size calculation was performed for this secondary analysis. The sample size was determined by the number of eligible patients enrolled in the parent ChTUIA cohort during the study period.

Statistical analyses were conducted using SPSS (version 24.0; Chicago, IL, USA). Continuous variables with a normal distribution are presented as mean ± standard deviation; otherwise, they are presented as median and interquartile range (IQR). Categorical variables are presented as numbers and percentages. Differences in continuous variables were compared using the Student’s t-test or Wilcoxon rank-sum test, as appropriate, and differences in categorical variables were compared using the chi-square test or Fisher’s exact test.

Logistic regression analysis was performed to compare the primary and secondary outcomes between the LSS and FSS groups. For CNS infection, incision infection, and mRS outcome, odds ratios (ORs) were calculated; for the Face-Q score, ordinal logistic regression was performed and ordinary ORs were reported. Results are presented as ORs (or ordinary ORs) with 95% confidence intervals (CIs).

Missing data were handled using a complete-case approach, and no imputation was performed. No formal sensitivity analyses were performed.

Subgroup analyses for CNS infection were performed according to age (≥ 50 years or < 50 years), sex (male or female), diabetes mellitus, dyslipidemia, aneurysm size (< 7 mm or 7–10 mm), aneurysm site (Acom, MCA, or ICA), surgery duration > 3 h (yes or no), and intraoperative bleeding > 500 mL (yes or no). A two-sided *P* < 0.05 was considered statistically significant.

## Results

### Baseline information

The current study reviewed 6712 patients out of 8005 patients. One thousand one hundred thirty five patients were excluded due to exclusion criteria and 158 due to lost to follow-up or withdrawal from follow-up. 3101 (46.2%) patients were male and the median age was 59 (IQR, 52–66) years. 359 (5.3%) patients had diabetes mellitus and 664 (9.9%) had dyslipidemia. One thousand one hundred four (16.4%) patients were current smokers. Eight hundred seventeen (12.2%) patients received LSS and 5895 (87.8%) received FSS. Of all treated UIAs, 880 (34.1%) UIAs sited in ICA, 2166 (49.1%) in ACA/Acom, and 3148 (16.8%) in MCA. Six thousand one hundred five (91.0%) UIAs were treated by clipping and 607 (9.0%) by wrapping. 

**Fig. 1:**
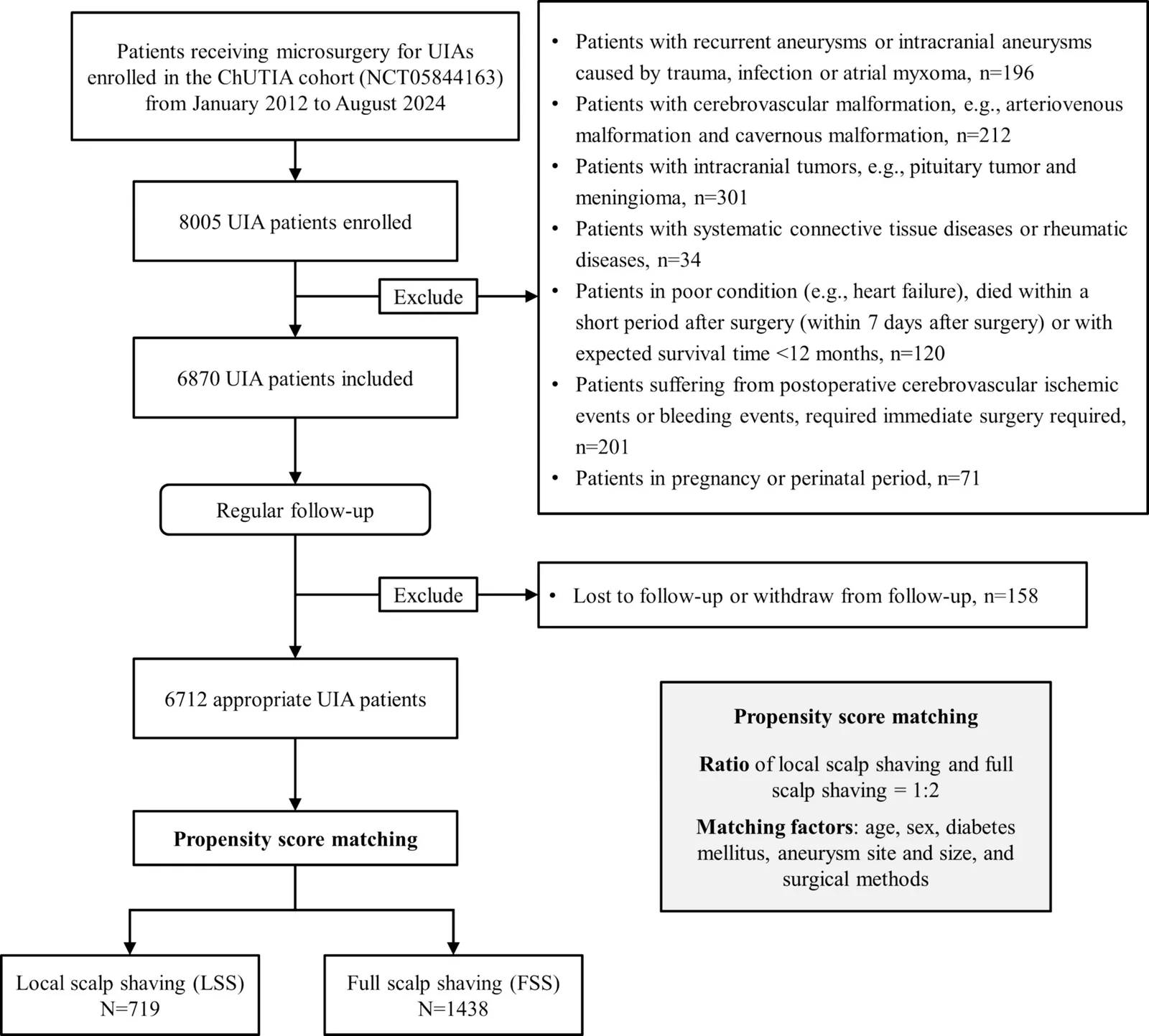
Flowchart of patient enrollment. The current study included 2157 UIA patients receiving microsurgical treatment, out of 8005 patients, after propensity score matching. Of them, 719 patients received LSS and 1438 received FSS. LSS, local scalp shaving; FSS, full scalp shaving

**Fig. 2:**
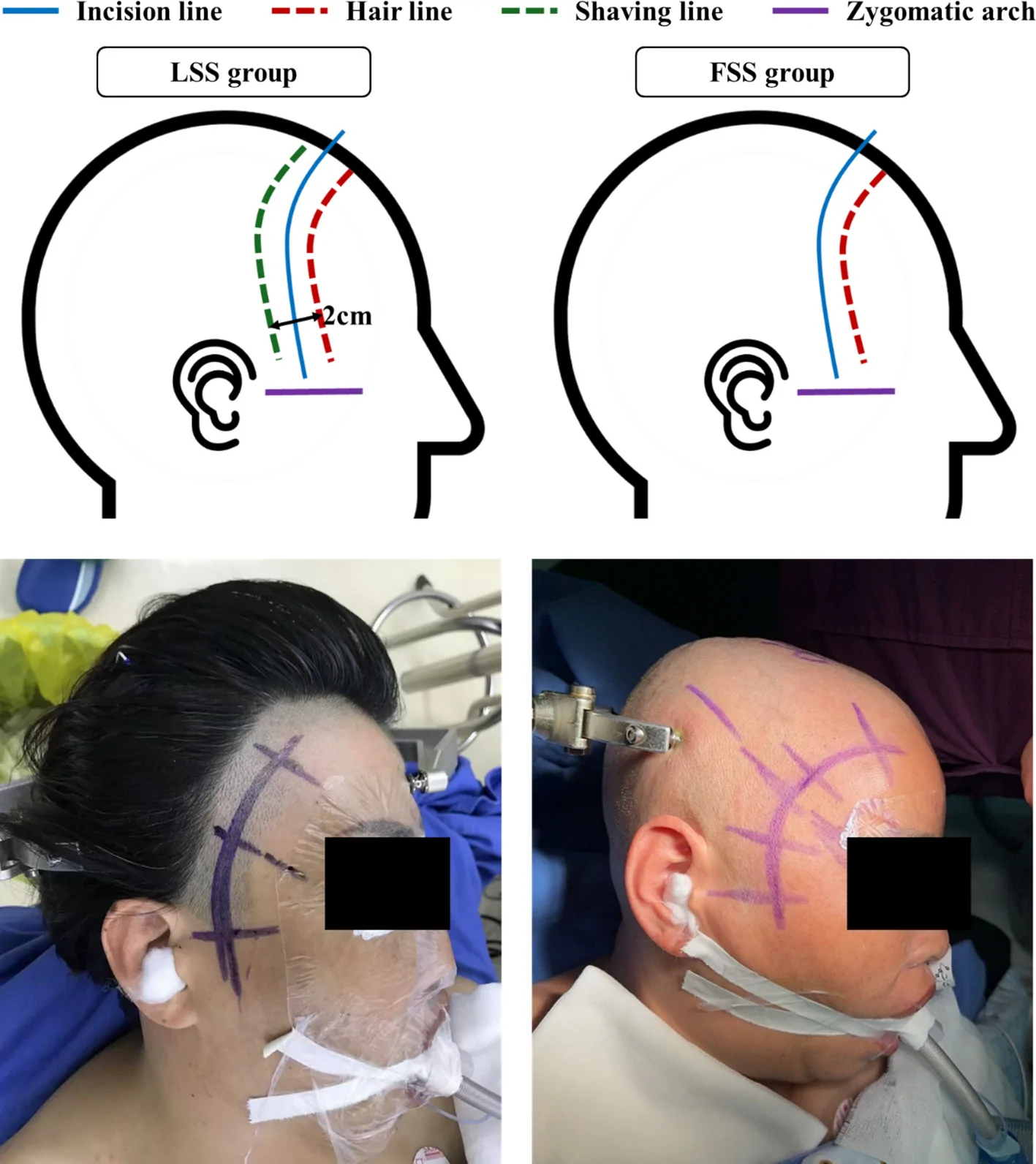
Representative cases in the LSS group and FSS group. For patients in the LSS group, the scalp shaving range is from hairline to 2 cm after this line. For patients in the FSS group, all hair is shaved for scalp preparation. LSS, local scalp shaving; FSS, full scalp shaving

The propensity score matching process included 2157 patients in the final analysis (Fig. 1). The information of all included patients is given in Table 1. After matching, covariate balance was generally acceptable, with most standardized mean differences (SMDs) below 0.1. Seven hundred nineteen patients received LSS and 1438 patients received FSS. The comparison of the characteristics between the LSS group and the FSS group found no difference in sex, age, smoking history, hypertension, diabetes mellitus, dyslipidemia, ICCD, aneurysm site and size, surgical methods, surgery duration, intraoperative bleeding and transfusion, and mRS on admission (all *P* > 0.05). The illustration and surgical findings of representative patients in the LSS group and FSS group are shown in Fig. 2.

**Table 1:** Demographic and clinical information of included UIA patients

Characteristics	LSS *N* = 719	FSS *N* = 1438	*P* value	SMD
Sex, n (%)			0.478	0.032
Male	295 (41.0%)	613 (42.6%)		
Female	424 (59.0%)	825 (57.4%)		
Age, years, median (IQR)	59 (51–66)	60 (52–67)	0.123	0.090
Age > 50 years, *n* (%)	570 (79.3%)	1156 (80.4%)	0.542	0.028
Smoking history			0.399	0.061
Current	106 (14.7%)	219 (15.2%)		
Ever	139 (19.3%)	244 (17.0%)		
No	474 (66.0%)	975 (67.8%)		
Comorbidities, *n* (%)				
Hypertension	176 (24.5%)	406 (28.2%)	0.064	0.085
Diabetes mellitus	39 (5.4%)	69 (4.8%)	0.530	0.028
Dyslipidemia	39 (5.4%)	92 (6.4%)	0.372	0.041
ICCD	53 (7.4%)	117 (8.1%)	0.534	0.029
Aneurysm site, *n* (%)			0.155	0.107
ICA	250 (34.8%)	485 (33.7%)		
ACA/Acom	367 (51.0%)	693 (48.2%)		
MCA	102 (14.2%)	260 (18.1%)		
Aneurysm size, *n* (%)			0.066	0.084
< 7 mm	418 (58.1%)	776 (54.0%)		
7-10 mm	301 (41.9%)	662 (46.0%)		
Surgical methods, *n* (%)			0.186	0.061
Clipping	672 (93.5%)	1321 (91.9%)		
Wrapping	47 (6.5%)	117 (8.1%)		
Surgery duration, min, median (IQR)	206 (172–257)	200 (166–255)	0.108	0.093
Surgery duration > 3 h, *n* (%)	225 (31.3%)	437 (30.4%)	0.668	0.020
Intraoperative bleeding > 500 ml, n (%)	130 (18.1%)	246 (17.1%)	0.574	0.026
Intraoperative transfusion, *n* (%)	114 (15.9%)	245 (17.0%)	0.487	0.032
mRS on admission, *n* (%)			0.446	0.036
0	707 (98.3%)	1407 (97.8%)		
1	12 (1.7%)	31 (2.2%)		

^†^Significant

*UIA* unruptured intracranial aneurysm, *LSS* local scalp shaving, *FSS* full scalp shaving, *ICCD* ischemic cerebrovascular or cardiovascular disease, *ICA* internal carotid artery, *ACA* anterior cerebral artery, *Acom* anterior communicating artery, *MCA* middle cerebral artery, *mRS* modified Rankin scale

### Primary and secondary outcomes

**Fig. 3:**
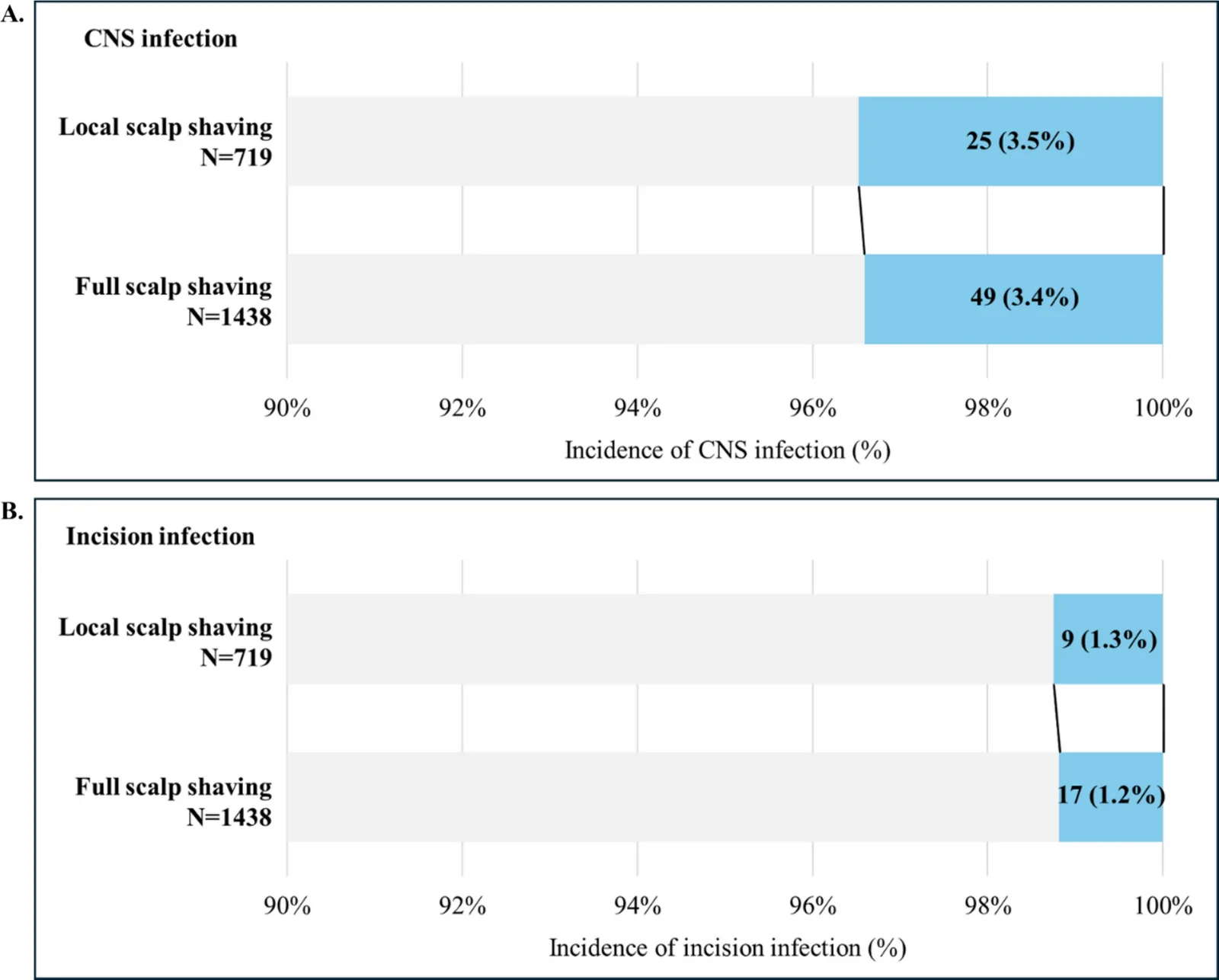
Incidence of infection in the LSS group and FSS group. **A** The incidence of CNS infection in the LSS group and FSS group. No significance was found in the incidence of CNS infection between the LSS group and the FSS group (25 of 719 [3.5%] *vs.* 49 pf 1438 [3.4%]; OR, 0.98; 95%CI, 0.60–1.60; *P* = 0.933). **B** The incidence of incision infection in the LSS group and FSS group. No significance was found in the incidence of CNS infection between the LSS group and the FSS group (9 of 719 [1.3%] *vs.* 17 pf 1438 [1.2%]; OR, 0.94; 95%CI, 0.42–2.13; *P* = 0.889). LSS, local scalp shaving; FSS, full scalp shaving; CNS, central nervous system; OR, odds ratio

Table 2 shows the incidence of primary and secondary outcomes. 25 of 719 (3.5%) patients in the LSS group and 49 of 1438 (3.4%) patients in the FSS group suffered from CNS infection after surgery. No increasing risk was found in the LSS group compared with the FSS group (OR, 0.98; 95%CI, 0.60–1.60; *P* = 0.933; Fig. 3). As for the secondary outcomes, no significant difference was found in the incision infection (9 of 719 [1.3%] *vs.* 17 of 1438 [1.2%]; OR, 0.94; 95%CI, 0.42–2.13; *P* = 0.889) and 180-day mRS score of 0–1 (692 of 719 [96.2%] *vs.* 1385 of 1438 [96.3%]; OR, 0.98; 95%CI, 0.61–1.57; *P* = 0.936). The median Face-Q score at 180 days was 4 (IQR, 3–5) in the LSS group and 2 (IQR, 0–3) in the FSS group. Patients in the LSS group had a higher Face-Q score on the 180th day after surgery compared with patients in the FSS group (ordinary OR, 0.43; 95%CI, 0.40–0.47; *P* < 0.001).

**Table 2:** Outcomes in LSS group and FSS group

Outcomes	LSS *N* = 719	FSS *N* = 1438	ORs (95%CI)	*P* value
90-day CNS infection, *n* (%)	25 (3.5%)	49 (3.4%)	0.98 (0.60–1.60)	0.933
90-day incision infection, *n* (%)	9 (1.3%)	17 (1.2%)	0.94 (0.42–2.13)	0.889
180-day mRS score, *n* (%)			0.98 (0.61–1.57)	0.936
0–1	692 (96.2%)	1385 (96.3%)		
2–5	27 (3.8%)	53 (3.7%)		
180-day Face-Q score, median (IQR)	4 (3–5)	2 (0–3)	0.43 (0.40–0.47)	< 0.001

*LSS* local scalp shaving, *FSS* full scalp shaving, *CNS* central nervous system, *mRS* modified Rankin scale, *OR* odds ratio

**Fig. 4:**
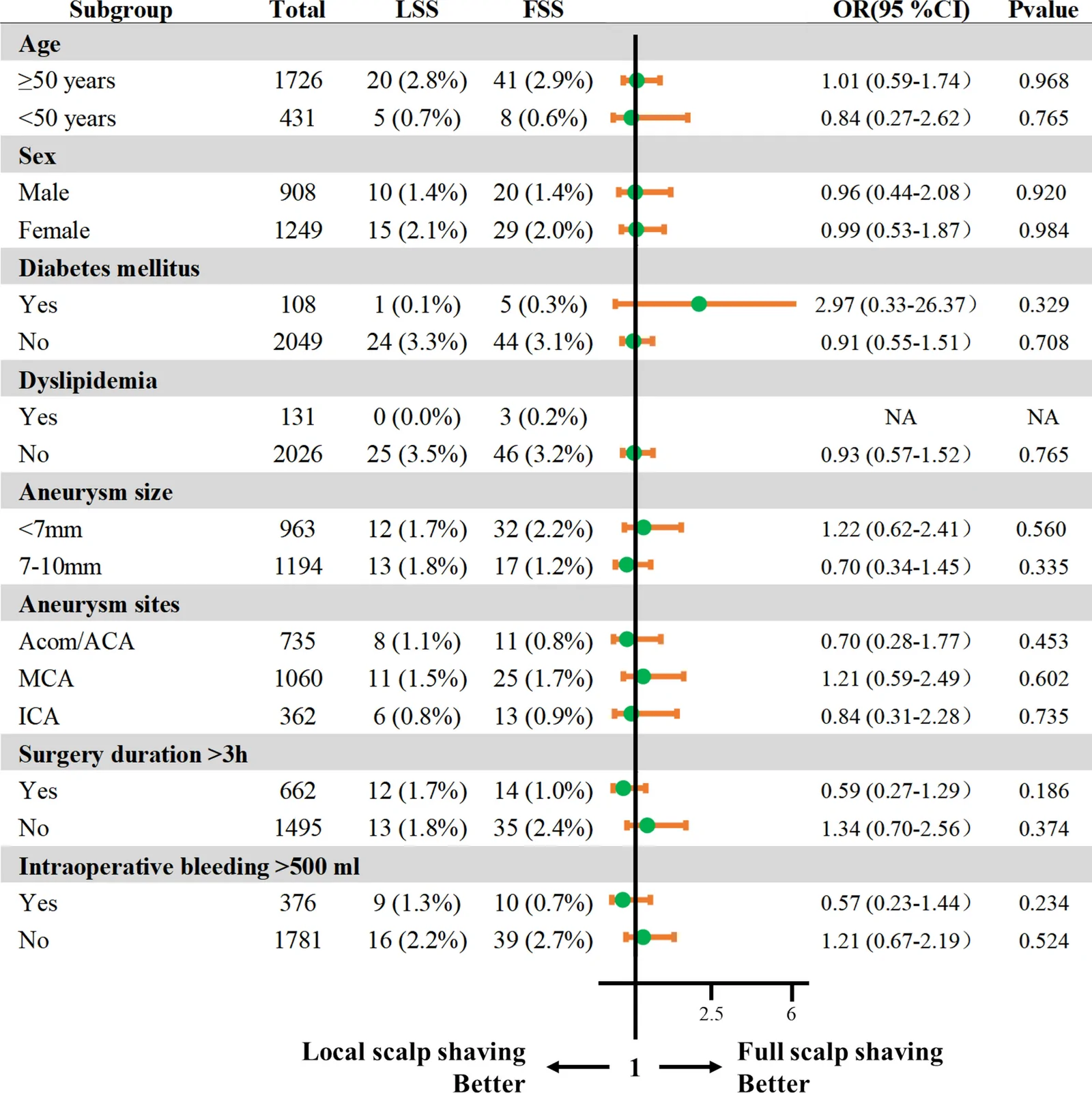
Subgroup analysis of scalp shaving method for CNS infection. LSS, local scalp shaving; FSS, full scalp shaving; ICA, internal carotid artery; ACA, anterior cerebral artery; Acom, anterior communicating artery; MCA, middle cerebral artery. NA denotes not applicable

Subgroup analysis showed that there was no significant difference in the incidence of CNS infection between the FSS group and LSS group based on all prespecified subgroups (all *P* > 0.05, Fig. 4).

## Discussion

An appropriate scalp shaving protocol may improve postoperative quality of life in patients undergoing microsurgical treatment for UIAs in the anterior circulation without increasing the risk of postoperative complications, including infection. In this propensity score-matched analysis nested within a prospective multicenter cohort, we found no significant differences between the LSS and FSS groups in 90-day CNS infection, 90-day incision infection, or 180-day functional outcome measured by the mRS, including in prespecified subgroups such as older patients, patients with diabetes mellitus, and those with surgery duration > 3 h. In contrast, patients in the LSS group had significantly better postoperative appearance-related satisfaction, as reflected by higher 180-day Face-Q scores. Taken together, these findings suggest that LSS is a feasible and patient-centered preoperative scalp preparation strategy for microsurgical treatment of anterior circulation UIAs, with no apparent increase in infection risk and with better postoperative quality of life.

From a biological perspective, the relationship between scalp shaving and postoperative infection risk is likely multifactorial. Traditionally, hair has been regarded as a potential source of contamination, leading to routine preoperative full scalp shaving [1]. However, hair itself may be less important than the integrity of the scalp barrier and the quality of perioperative sterile preparation. Razor- or clipper-related microtrauma may disrupt the skin barrier, alter the local microbiota, and facilitate bacterial colonization, thereby potentially increasing the risk of wound contamination [9–11]. At the same time, postoperative infection is also influenced by multiple perioperative factors, including antiseptic preparation, sterile draping, wound closure quality, operative duration, cerebrospinal fluid leakage, drain use, and perioperative antibiotic management. Therefore, it is biologically plausible that reducing the shaving area does not increase infection risk when standard sterile preparation is adequately maintained.

Our findings are generally consistent with the broader published evidence suggesting that extensive preoperative scalp shaving does not confer a clear infection-related advantage in neurosurgery. A systematic review including 21 studies found that preoperative shaving was not superior to no shaving in preventing postoperative wound infection and might even increase infection risk [11]. Another systematic review reported similar conclusions and highlighted the cosmetic and psychological value of minimizing hair removal [12]. In addition, more recent studies in cranial surgery have shown that hair-sparing or reduced-shaving approaches are feasible and are not associated with worse postoperative infection outcomes [13, 14]. Compared with the existing literature, the present study provides additional clinically relevant evidence from a large multicenter cohort focused specifically on microsurgical treatment of anterior circulation UIAs. Moreover, by simultaneously evaluating both infection-related outcomes and patient-reported quality of life, our study extends the discussion beyond surgical safety alone and supports a more patient-centered perspective on preoperative scalp preparation.

The significantly higher 180-day Face-Q scores observed in the LSS group further support the clinical value of minimizing scalp shaving when feasible. Hair is a highly visible component of personal appearance and social identity, and full scalp shaving may contribute to emotional distress, impaired body image, and delayed social reintegration [3, 4, 15–17] . In this context, LSS may improve patients’ postoperative experience not by altering neurological recovery, but by reducing the psychosocial burden associated with surgery. This distinction is important, because a preparation strategy that preserves patient dignity and appearance without compromising safety may be especially relevant in elective surgery for unruptured aneurysms.

Several limitations should be acknowledged. First, although propensity score matching was used to reduce baseline imbalance, residual confounding cannot be fully excluded because of the observational design. Some potentially relevant perioperative variables, such as incision length, scalp closure technique, cerebrospinal fluid leakage, drain use, perioperative antibiotic details, and scalp thermal injury during electrocautery, were not available for adjustment and may have influenced infection risk or wound healing. Second, although prespecified definitions of LSS and FSS were used across participating centers, minor center-level variations in local implementation may still have existed. Third, because this study included only a Chinese population, the generalizability of the findings to other healthcare settings and patient populations should be interpreted with caution. Fourth, although we assessed infection, functional outcome, and appearance-related satisfaction, other relevant outcomes, such as long-term cosmetic satisfaction, scar-related perception, and delayed wound complications, were not evaluated. Finally, the Face-Q score is a patient-reported outcome and may therefore be subject to subjective bias despite the use of a standardized instrument.

## Conclusion

LSS is an appropriate preoperative scalp shaving protocol for patients receiving microsurgical treatment of UIAs in the anterior circulation, which doesn’t have the additional risk of infection and could improve the quality of life after surgery. 

## Supplementary Information

Supplementary Material 1.

## Data Availability

Anonymized data that support the findings of this study are available from corresponding author on reasonable request from qualified investigators.

## Abbreviations

UIA: Unruptured intracranial aneurysms
LSS: Local scalp shaving
FSS: Full scalp shaving
CNS: Central nervous system
ICCD: Ischemic cerebrovascular or cardiovascular disease
ICA: Internal carotid artery
ACA: Anterior cerebral artery
Acom: Anterior communicating artery
MCA: Middle cerebral artery

## References

[1] Husted H, Gromov K, Malchau H, Freiberg A, Gebuhr P, Troelsen A. Traditions and myths in hip and knee arthroplasty. Acta Orthop. 2014;85(6):548–55. 10.3109/17453674.2014.971661. 25285615 PMC4259040

[2] Pan A, Ambrosini L, Patroni A, et al. Adherence to surgical site infection guidelines in Italian cardiac surgery units. Infection. 2009;37(2):148–52. 10.1007/s15010-008-7474-8. 19308319

[3] Palese A, Moreale R, Noacco M, et al. Post-operative shampoo effects in neurosurgical patients: a pilot experimental study. Surg Infect (Larchmt). 2015;16(2):133–8. 10.1089/sur.2014.028. 25671762

[4] Shimamura N, Ohkuma H. How can we manage skin incision and craniotomy for avoidance of postoperative esthetic concern. No Shinkei Geka. 2021;49(1):31–40. 10.11477/mf.1436204358. 33494049

[5] Bekar A, Korfali E, Doğan S, Yilmazlar S, Başkan Z, Aksoy K. The effect of hair on infection after cranial surgery. Acta Neurochir (Wien). 2001;143(6):533–7. 10.1007/s007010170057. 11534669

[6] Luther E, Berry K, McCarthy D, et al. Hair-sparing technique using absorbable intradermal barbed suture versus traditional closure methods in supratentorial craniotomies for tumor. Acta Neurochir (Wien). 2020;162(4):719–27. 10.1007/s00701-020-04239-3. 32002670

[7] Richards A, Zaben M, Patel C, Leach P. The need for hair removal in paediatric brain tumour surgery? Br J Neurosurg. 2021;38(2):346–8. 10.1080/02688697.2021.1872777. 33455445

[8] Zheng K, Wen Z, Wang K, et al. Treatment strategies for unruptured intracranial aneurysms in the Chinese population: China Treatment Trial for Unruptured Intracranial Aneurysm (ChTUIA). Chin Neurosurg J. 2025;11(1):8. 10.1186/s41016-025-00394-7. Published 2025 Apr 3. 40181400 PMC11969722

[9] Jubbal KT, Zavlin D, Echo A. Optimizing exposure for the occipital nerve in migraine surgery while maintaining hair length. Plast Reconstr Surg Glob Open. 2017;5(11):e1518. 10.1097/gox.0000000000001518. 29263947 PMC5732653

[10] Mann M, Wright CH, Jella T, et al. Cranial surgical site infection interventions and prevention bundles: a systematic review of the literature. World Neurosurg. 2021;148:206-219.e4. 10.1016/j.wneu.2020.12.137. 33412319

[11] Broekman ML, van Beijnum J, Peul WC, Regli L. Neurosurgery and shaving: what’s the evidence? J Neurosurg. 2011;115(4):670–8. 10.3171/2011.5.JNS102003. 21721875

[12] Sebastian S. Does preoperative scalp shaving result in fewer postoperative wound infections when compared with no scalp shaving? A systematic review. J Neurosci Nurs. 2012;44(3):149–56. 10.1097/jnn.0b013e31825106d2. 22555352

[13] Tokimura H, Tajitsu K, Tsuchiya M, et al. Cranial surgery without head shaving. J Craniomaxillofac Surg. 2009;37(8):477–80. 10.1016/j.jcms.2009.06.003. 19604702

[14] Plaha P, Patel N, Gill S. Implantation of deep brain stimulation electrodes in unshaved patients. JNS. 2003;99(1):207–8; author reply 208-9. 10.3171/jns.2003.99.1.0207. 12854769

[15] Tang K, Yeh JS, Sgouros S. The influence of hair shave on the infection rate in neurosurgery. Pediatr Neurosurg. 2001;35(1):13–7. 10.1159/000050379. 11490185

[16] Winston KR. Hair and Neurosurgery. Neurosurg. 1992;31(2):320???329. 10.1097/00006123-199208000-00018. 1513437

[17] Liu WJ, Duan YC, Chen MJ, Tu L, Yu AP, Jiang XL. Effectiveness of preoperative shaving and postoperative shampooing on the infection rate in neurosurgery patients: a meta-analysis. Int J Nurs Stud. 2022;131:104240. 10.1016/j.ijnurstu.2022.104240. 35490453

