# Supplementary material for: Infection and quality of life after local or full scalp shaving in microsurgical treatment for unruptured intracranial aneurysm sited in anterior circulation
Source: Chin Neurosurg J. 2026 Sep 16;12:31. doi: 10.1186/s41016-026-00449-3 (PMC13579903; doi:10.1186/s41016-026-00449-3)
Supplement: Supplementary file 1 [file 41016_2026_449_MOESM1_ESM.docx]

**Supplemental material for** “*Infection and quality of life after local or full scalp shaving in microsurgical treatment for unruptured intracranial aneurysm sited in anterior circulation*”

**Supplemental tables**

**Supplemental table 1. Face-Q questionnaire.**

| NO. | Questions |  | Points |
| --- | --- | --- | --- |
| 1 | If you feel your face looks natural | Yes | 1 |
|  |  | No | 0 |
| 2 | If you feel young inside then you look in the mirror | Yes | 1 |
|  |  | No | 0 |
| 3 | If it made you more confident | Yes | 1 |
|  |  | No | 0 |
| 4 | If you feel confident in new or old social situations | Yes | 1 |
|  |  | No | 0 |
| 5 | If you have any incision infection, pain, swelling, or bruising | Yes | -1 |
|  |  | No | 0 |
| 6 | If you have any facial numbness or difficulty in opening mouth | Yes | -1 |
|  |  | No | 0 |
| 7 | If you satisfy the process of care and your physician-in-charge | Yes | 1 |
|  |  | No | 0 |

Total Face-Q score is -2 to 5 points. The higher the Face-Q scores patients had, the more satisfied they were with their facial appearance after surgery.
